# Targeting the neural stem cells in subventricular zone for the treatment of glioblastoma: an update from preclinical evidence to clinical interventions

**DOI:** 10.1186/s13287-023-03325-4

**Published:** 2023-05-11

**Authors:** Sijia Li, Lihua Dong, Zhenyu Pan, Guozi Yang

**Affiliations:** 1 Jilin Provincial Key Laboratory of Radiation Oncology and Therapy, Department of Radiation Oncology and Therapy, The First Hospital of Jilin University, Changchun, 130021 China; 2 Department of Radiation Oncology, Huizhou Third People’s Hospital, Guangzhou Medical University, Huizhou, 516000 China

**Keywords:** Glioblastoma, Subventricular zone, Neural stem cells, Glioma stem cells, Radiation

## Abstract

**Background:**

Glioblastoma is one of the most common and aggressive adult brain tumors. The conventional treatment strategy, surgery combined with chemoradiotherapy, did not change the fact that the recurrence rate was high and the survival rate was low. Over the years, accumulating evidence has shown that the subventricular zone has an important role in the recurrence and treatment resistance of glioblastoma. The human adult subventricular zone contains neural stem cells and glioma stem cells that are probably a part of reason for therapy resistance and recurrence of glioblastoma.

**Main body:**

Over the years, both bench and bedside evidences strongly support the view that the presence of neural stem cells and glioma stem cells in the subventricular zone may be the crucial factor of recurrence of glioblastoma after conventional therapy. It emphasizes the necessity to explore new therapy strategies with the aim to target subventricular zone to eradicate neural stem cells or glioma stem cells. In this review, we summarize the recent preclinical and clinical advances in targeting neural stem cells in the subventricular zone for glioblastoma treatment, and clarify the prospects and challenges in clinical application.

**Conclusions:**

Although there remain unresolved issues, current advances provide us with a lot of evidence that targeting the neural stem cells and glioma stem cells in subventricular zone may have the potential to solve the dilemma of glioblastoma recurrence and treatment resistance.

## Background

Glioblastoma (GBM) is one of the most common and aggressive primary central nervous system malignancies in adults. The standard therapies, surgery combined with adjuvant treatment, yield poor prognosis. New therapeutic strategies include tumor-treating fields and immune checkpoint inhibitors have been explored to improve clinical outcomes in recent years [1]. However, even after aggressive treatment, both local and distant tumor recurrence are usually inevitable [2]. The rate of distant recurrence has even been reported as high as 43% [3]. In addition, treatment resistance is also a critical factor leading to the poor prognosis of GBM patients [4]. Exploring the cue for the recurrence and treatment resistance may provide new treatment strategy for GBM.

Emerging data support the hypothesis that the presence of a small population of glioma stem cells (GSCs) was a part of reason for therapy resistance and recurrence of GBM [5, 6]. GSCs are mainly present in the tumor mass but have also been detected in the subventricular zone (SVZ) [7], which is a neuroprimitive zone after birth and also contains resident neural stem cells (NSCs) that may contain origin cells of human GBM driver mutation [8, 9]. Numerous retrospective studies have found that patients with GBMs contacting the SVZ have worse clinical outcomes and more aggressive patterns of recurrence [10–12]. Moreover, GBMs with SVZ involvement have been demonstrated to show a higher propensity to chemotherapy and radiation resistance [13]. These findings have indicated that SVZ as a NSCs niche may be a critical factor for GBM recurrence and treatment resistance.

Therefore, whether SVZ can be used as a potential therapeutic target for GBM has attracted researchers' attention. This review clarifies the clinical relevance of SVZ in GBM, introduces the role of SVZ NSCs niche in GBM, lists the preclinical evidence and clinical data of SVZ as a potential therapeutic target for GBM, and makes a certain prospect of clinical application of SVZ as a therapeutic target for GBM.

## The clinical relevance of SVZ in GBM

SVZ is a 3–5 mm thick area, situated on the outside wall of each lateral ventricle of the vertebrate brain [14]. It is present in both the embryonic and adult brain. The SVZ consists of three layers. The first layer consists of a single layer of ependymal cells. The second layer, called subcellular space, is composed of ependymal and astrocytic cells. The third layer consists of glial fibrillary acidic protein-positive astrocyte-like neural precursor cells (NPCs) and CD133-positive NSCs [15]. It is now believed that GBM originates from the accumulation of somatic mutations in NSCs in the SVZ [16, 17]. SVZ has been shown to offer to GSCs a particular microenvironment participating in their resistance to chemoradiotherapy [18]. GSCs could migrate from the tumor mass toward the SVZ, where they could escape therapies and be involved in GBM recurrences [18, 19]. Since the notion that human gliomas may arise from SVZ NSCs was first proposed in the first half of the twentieth century [20], the accumulating clinical data suggested that the relationship of GBM to the SVZ was associated with clinical outcome of patients.

**Fig. 1:**
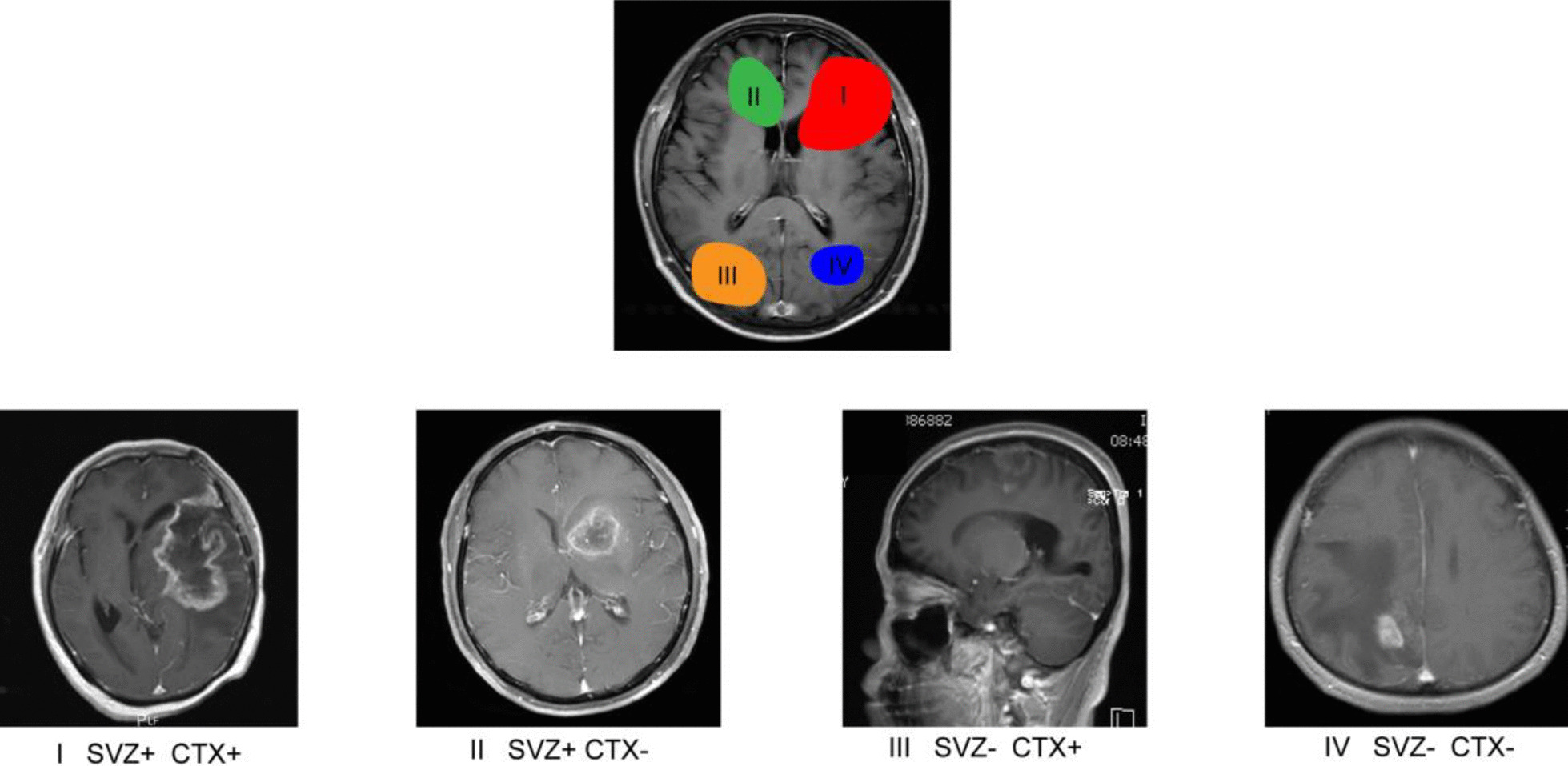
GBM was divided into 4 groups according to the relationship with SVZ and cortex (CTX). The first group was exposed to SVZ and the CTX; the second group was exposed to SVZ but not the CTX; the third group was exposed to the CTX but not the SVZ; the fourth group did not touch these two parts. Schematic created with Adobe Illustrator

In some previous studies, GBM was categorized according to the relationship of GBM to the SVZ. Four subtypes were determined (Fig. 1), and the studies’ results suggested that GBM contacting the SVZ tend to show multifocal relapse, earlier recurrence, and decreased overall survival (OS) [10, 11, 20, 21]. Weinberg et al. [22] suggested that GBM often recurs in SVZ, which can be SVZ close to the primary site or SVZ far away, regardless of whether the primary tumor is contacted to SVZ. And several studies have shown that SVZ exposure may be an independent risk factor for poorer prognosis in GBM patients [12, 19, 23–27].

However, there are also a few studies showing different results. In a study by Kimura et al., the results suggested that GBM patients’ location relative to SVZ does not predict patterns of tumor recurrence and/or progression. Yet it should be noted that their study had such limitations: the total sample size was small, the number of samples in contact with SVZ was limited, and only contrast agent enhancement was used as the classification standard, without considering magnetic resonance imaging (MRI) Spectrum and MRI Perfusion [28]. Since that, it was possible that the contradictory conclusions drawn from this study may be related to the limitations of the study mentioned above.

## The role of SVZ neural stem cell niche in GBM

Since the beginning of this century, cancer stem cells (CSCs) have been successfully identified in several solid tumors, including breast, colorectal and brain cancers (including GBM). In GBM, CSCs are specifically named GSCs. GSCs exhibit resistance to chemotherapy and radiotherapy, while contributing to invasion, angiogenesis, and tumor recurrence [29]. SVZ is the largest adult neural stem cell niche [30]. NSCs and tumor cells share a common pattern of behavior and movement, and studies have shown that GSCs in GBM are derived from adult NSCs [8, 15]. The SVZ microenvironment also induces GBM to dedifferentiate into a more stem-like state [31]. Therefore, tumors exposed to SVZ exhibit faster progression and more aggressive clinical behavior [19, 25, 32].

### The relationship among SVZ, NSCs, GBM, and GSCs

Studies have shown that SVZ NSCs have multilineal potential like GSCs, such as strong self-renewal ability, proliferation ability and migration ability. Some molecular evidence suggests that GBM results from the migration of mutant NSCs from SVZ. In addition to the apparent functional overlap, the similarity of many gene expression patterns including CD133, Sox10, Nestin, Musashi, GFAP, and Olig1/2 highlight the shared molecular programming between NSCs and GSCs [29]. Through deep sequencing of isocitrate dehydrogenase wild-type GBM patient samples and normal SVZ tissue, researchers observed similar expression of driver mutations in both the SVZ and patient matched-tumor tissue [9]. There are some differences between this regulation and GSCs’. Compared with NSCs, GSCs are self-sufficient in providing growth signals, resisting growth inhibition, avoiding programmed apoptosis, having unlimited replication potential, maintaining angiogenesis, and invading surrounding tissues [29]. There are also similarities and differences between the two at the molecular level. Hira et al. confirmed by fluorescence immunohistochemistry and image analysis that CD133 and COX2 were expressed in both GSCs and NSCs, while CD9 was only expressed in GSCs, verifying that CD133 and COX2 are biomarkers of GSCs and NSCs, and CD9 is a selective GSCs biomarker. It is demonstrated for the first time that both NSCs and GSCs are specifically localized in the SVZ at a distance from the GBM [15]. Therefore, it is crucial for researchers to understand the similarities and differences between GSCs and NSCs in order to find new targeted therapies. Comparison of similarities can help better understand the molecular mechanism driving GBM formation, while comparison of differences can help identify unique molecular vulnerability, which may be more effective in eliminating GBM potential recurrence and reducing the risk of GBM recurrence.

### The role of SVZ neural stem cell in GBM development

SVZ NSCs play a role in tumorigenesis, progression, and recurrence of GBM. Studies have shown that the original gene mutations of human GBM-derived cells may be derived from the NSCs of SVZ, and GBM may result from an increase in NSCs gene mutations [8, 9, 33, 34]. Several studies subsequently provided molecular evidence that the NSCs in SVZ with mutations in the tumor protein P53 or IDH1 genes can lead to uncontrolled proliferation and tumorigenesis [8, 9]. Studies have proved that the origin cells of GBM have the characteristics of stem cells and are the source of tumor recurrence [35]. Therefore, NSCs in SVZ may be involved in the development of primary and recurrent GBM [36, 37].

Due to the migration ability of GSCs and the unique environment of SVZ (the vascular system of SVZ is richer than that of other brain regions, which can provide sufficient nutrition to tumor cells [7]), treatment-resistant GSCs are easy to migrate to and colonize in SVZ. As a haven of tumor cells, SVZ hinds the complete clearance of GSCs and promotes tumor progression.

As mentioned in the first part of this paper, some current clinical studies have found that GBM exposed to SVZ is more likely to relapse [20]. This may be due to the fact that NSCs in SVZ can leave the niche and migrate over long distances to promote tumor relapse, and GBM exposed to SVZ are closely related to cerebrospinal fluid, which circulates and spreads tumor cells to distant locations [25]. Studies have shown that GBM patients exposed to SVZ have a higher expression rate of CD133 (a GBM biomarker related to radiation resistance expressed by both NSCs and GSCs) than those not exposed [36, 38–40]. Therefore, CD133 expression by NSCs in SVZ may be a factor in the high recurrence and poor prognosis of GBM.

### The SVZ neural stem cell microenvironment in GBM

The NSC niche is a broad microenvironment that hosts cell–cell and cell-microenvironment interactions [41]. Recent studies have shown that GBM patients who contact the SVZ of the lateral ventricle have lower survival rates than those who contact the subgranular zone, corpus callosum, or cortex [33]. The study found that the malignant degree of proximal ventricular GBM may not be an intracellular factor, but a product of SVZ microenvironment [42].

**Fig. 2:**
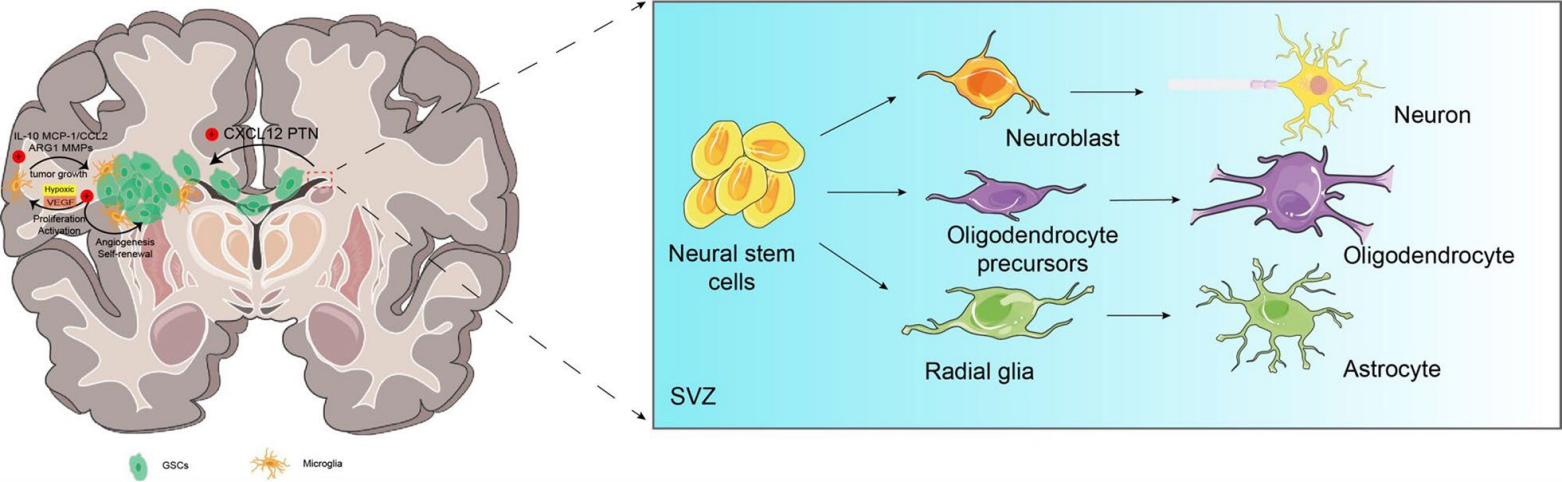
Relationships among GSCs, NSCs and microglia and physiological outcomes of NSCs. NSCs in SVZ induce high-grade glioma to invade SVZ by secreting specific chemokines like CXCL12, some proteins like PTN that regulate cell migration. And they play a key role in migration of GSCs from tumor masses to SVZ. Microglia also play an important role in the microenvironment of GSCs and NSCs. SVZ is the largest repository of adult NSCs. Under physiological conditions, NSCs differentiate into neurons, oligodendrocytes, and astrocytes. Clinically, NSCs of SVZ transformed into oligodendrocytes are more likely to develop into GBM. Schematic created with Adobe Illustrator

NSCs in SVZ induce high-grade glioma to invade SVZ by secreting specific chemokines and other proteins that regulate cell migration, of which CXCL12 plays a key role in migration of GSCs from tumor masses to SVZ [43, 44]. And Pleiotrophin (PTN) is considered as a potential target for glioma treatment. PTN protein is necessary for GBM to invade the NPCs of SVZ. The reduced invasion ability of GBM is not due to the loss of NPCs, but due to the reduced expression of PTN, which is abundant in SVZ NSCs [45]. High expression of epidermal growth factor receptor (EGFR) was associated with a higher distant recurrence rate [25]. EGFR mutations are present in about 40% of GBM patients which confer a proliferative advantage in NSCs and improve tumor cell survival [23, 36, 46] Microglia, the major macrophages of the CNS, are a key component in determining the fate of NSCs and play an important role in the microenvironment in which GBM progresses. In tumor masses, microglia have native activity and can stimulate tumor growth by several cytokines and chemokines, such as IL-10, monocyte chemoattractant protein-1 (MCP-1/CCL2), some metalloproteinases (MMPs), and ARG1. The hypoxic environment is also associated with tumor origin. Vascular endothelial growth factor (VEGF) induces the proliferation and activation of microglia and promotes self-renewal of GSCs under hypoxic conditions [47] (Fig. 2). All above indicate that the recurrence of GBM is closely related to the SVZ microenvironment. These results provide experimental evidence for GBM to invade the SVZ. Therefore, targeting the interaction between GBM and SVZ NSCs could represent a new strategy to reduce the malignant potential of SVZ NSCs and limit the progression of glioma.

## Preclinical and clinical evidence of SVZ as the potential therapeutic target for GBM

### Preclinical data of SVZ as the potential therapeutic target for GBM

In vivo and in vitro experiments support that inactivation of tumor suppressor genes in NSCs is a necessary condition for GBM induction. Although the role of NPCs in humans is unclear, NSCs/NPCs have shown glioma-tropism in many animal studies and have shown antitumor effects in some studies, improving survival in animal models [48–52]. (Specific studies are shown in Table 1.) Studies have shown that GBM can be induced from cells in the SVZ in animal models [53]. Rohrer Bley et al. [54] reviewed 32 cases of primary glioma in dogs between 2015 and 2020 and found that variables associated with increased risk of tumor progression included greater GTV and ventricular invasion and a tendency for tumor recurrence after exposure to SVZ. And tumors contacting the SVZ are more aggressive and invasive than those originating in other regions. Another study has shown that microglia are key components in determining the fate of NSCs. In animal models, the antiphagocytic protein CD47 on the cell surface of microglial tumor masses expression of its proto-oncophenotype and transforms it into a potential weapon to block the progression of GBM. Therefore, tumor-associated microglia have proved to be a key therapeutic target in GBM [47].

**Table 1:** Preclinical evidence of SVZ as a therapeutic target for GBM

Researcher	Object of study	Size of study	Methods	Result	References
Carla et al.	Dogs	32	Radiotherapy	Contacting the SVZ is more malignant and increases the risk of GBM progression	[54]
An et al.	CELLS and nude mice	–	U251 and normal NSCs co-culture	Normal NSCs possessed an anti-glioma property	[55]
Li et al.	Cells	–	U87 and normal NSCs/NPCs co-culture	NSCs/NPCs had inhibitory effects on the growth and invasion of U87 glioma in vitro	[56]
Ehtesham et al.	Cells and mice	–	Stereotactically inoculated co-cultured cells in the right corpus striatum	IL-12-secreting NSCs prolong survival	[59]
Benedetti et al.	Mice	–	Stereotactically inoculated co-cultured cells in the left striatum	IL-4- secreting NSCs had anti-tumor effect	[61]

*SVZ* subventricular zone, *GBM* glioblastoma, *NSCs* neural stem cells, *NPCs* neural precursor cells

Since current targeted therapy cannot penetrate the blood brain barrier and hard-to-reach GBM core, NSCs have been used to load therapeutic molecules for targeted therapy of GBM [33]. After intracranial or intravascular implantation, NSCs can target tumor cells through normal tissues and widely distribute in the tumor bed. An et al. [55] confirmed that normal NSCs have direct anti-glioma properties by inhibiting the viability, proliferation, migration, and invasion of glioma cells through in vitro co-culture of normal NSCs with rat/human glioma cells (C6/U251) and subcutaneous injection of NSCs and U251 cell lines in nude mice. In another report, contact with NSCs conditioned medium containing U87 stem cells (a glioma cell line) showed a lower survival rate and proliferation of U87 cells, and no significant regulatory effect on astrocyte differentiation. In addition, the invasion and migration of U87 stem cells were also reduced [56]. These suggest that normal NSCs may play a direct role in GBM.

Genetic changes in NSCs can induce the production of antitumor compounds near tumors, one of which is immune modulator [57]. IL-12 is a known T cell stimulator that not only activates natural killer cells, but also induces T cells to differentiate into CD4 + T cells of the Th1 subtype [58]. Ehtesham et al. [59] have shown that injection of NSCs secreting IL-12 improves survival, which is associated with a higher degree of tumor invasion by CD4 + and CD8 + T cells. IL-4 has been shown to enhance recruitment of precursor T cells, thereby enhancing the immune response to tumors [60]. Benedetti et al. [61] demonstrated the efficacy of IL-4 as an antitumor cytokine and showed that specific introduction of IL-4 through NSCs improves survival compared to retrovirus transfer of IL-4. This suggests that the inherent antitumor activity of NSCs may be partly responsible for their improvement. Both methods succeeded in reducing the tumor burden and prolonging the survival time of mice.

### Clinical evidence of SVZ as the potential therapeutic target for GBM

In a retrospective study of 176 surgically resected patients with recurrent GBM, partial resection, SVZ exposure, and TERT C228 wild-type were found to be independent risk factors for recurrence in GBM patients [10]. SVZ as a potential target for radiotherapy intervention, while some have found improvement in progression-free survival (PFS) in GBM patients after inclusion of the ipsilateral SVZ (iSVZ) in the high-dose region, others have not found any association, and the current findings remain inconclusive [54]. (Clinical evidences are shown in Table 2.) Evers et al. were the first to find that bilateral doses of SVZ above 43 Gy significantly improved the median PFS. The study included 55 patients with grade III or IV GBM who had received radiotherapy at the University of California from February of 2003 to May of 2009. And they were followed up from one month after the end of radiotherapy until disease progression or death. They were divided into a high-dose group and a low-dose group based on a median periventricular dose of 43 Gy on both sides. This retrospective study confirmed that PFS was significantly higher in the high-dose group than in the low-dose group (15 months vs. 7.2 months), with a statistically significant difference (*P* = 0.028 < 0.05) [62]. This retrospective study was based on the hypothesis that the dose of the normal tissue stem cell niche in the adult brain affects the efficacy of radiotherapy in GBM patients. Due to the short survival of GBM patients, no side effects of irradiation to the periventricular region on normal tissues have been observed, so prospective experiments are needed to further explore the efficacy and toxicity of incorporating the periventricular region as an additional target volume into the treatment plan for patients with GBM. The results of a larger retrospective study were reported three years later by Lee et al. In their study, which included 173 patients with grade IV GBM at two centers, they found that when iSVZ radiation was greater than 59.4 Gy, the median PFS has statistical significance (12.6 months vs. 9.9 months, *p* = 0.042), as for OS, the ipsilateral high-dose of SVZ tended to improve survival, but was not statistically significant (25.8 months vs. 19.2 months, *P* = 0.173). For patients limited to grade IV GBM, the radiation dose of 43 Gy had no effect on PFS or OS [63]. This is not consistent with the results of previous studies, which may be since the fact that in Evers’ study 31% of the sample were grade III GBM patients, which received lower radiation dose (50–54 Gy), while grade IV GBM has a higher degree of malignancy, and 43 Gy may not be enough to eliminate the relatively radiation-resistant GSCs in SVZ of the grade IV patients. Neither of these studies considered O6-methylguanine-methyltransferase promoter status. Aggressive salvage therapy impairs the power to detect survival differences, so whether high-dose irradiation of the SVZ improves OS needs to be further verified by randomized trials. Chen’s study showed that iSVZ dose was greater than 40 Gy, both PFS and OS improved. In this retrospective study, 116 patients with GBM who underwent surgery, radiotherapy, and chemotherapy between 2006 and 2009 were included. PFS was significantly improved when the ipsilateral SVZ dose was greater than 40 Gy, and OS was improved in patients with gross total resection (GTR), but not in patients with biopsy or subtotal resection (STR) [52]. Iuchi et al. [64] found that low fractionation and high-dose radiotherapy (PTV1 = 68 Gy/8f) had satisfactory results in local control and survival of GBM. But long-term toxicities in this study occurred more frequently and earlier. Five patients developed symptomatic radiation necrosis requiring open surgery. Damage to the SVZ is closely related to patient survival. Hypofractionated radiation has a higher risk of SVZ damage and may increase the risk of neurocognitive sequelae, but it also has stronger damage to GSCs. It is controversial whether the irradiation dose of the SVZ should be increased to control GSCs or whether the region should be preserved to protect NSCs. A study has shown that when the iSVZ is included in CTV and the target dose reaches 58 Gy, the median OS is 16 months, which is greater than the median survival time of 14 months when SVZ is not irradiated [65].

**Table 2:** Clinical evidence of SVZ as a therapeutic target for GBM

Researcher	Size of experiment	Surgery	Chemotherapy	Radiotherapy	Result	References
Huang et al.	176 patients	+	Partly	+	SVZ contact (*P* = 0.008) was significantly associated with a shortened recurrence time	[10]
Evers et al.	55 patients		+ (except one)	External beam radiation therapy (except one)	Bilateral SVZ received greater than the median SVZ dose (43 Gy) had a significant improvement in PFS	[62]
Lee et al.	173 patients	+		+	High radiation therapy doses to ipsilateral SVZ remained an independent predictor of improved PFS but not of OS	[63]
Chen et al.	116 patients	+	+	Intensity-modulated radiation therapy (IMRT (60 Gy/30 f))	iSVZ dose was greater than 40 Gy, both PFS and OS improved in patients with GBM after GTR	[52]
Luchi et al.	single-institution prospective study	+	+	Hypofractionated high-dose IMRT	Hypofractionated radiation (PTV1 = 68 Gy/8f) had satisfactory results in local control and survival	[64]
Darázs et al.	41 patients	+		+	Higher mean dose (≥ 58 Gy) to the iSVZ2 had significantly better OS	[72]
Adeberg et al.	607 patients	68.5%	71.7%	28.3%	GBM close to the SVZ has decreased survival and a higher risk of multifocal or distant progression	[21]
Gupta et al.	40 patients	+	+	Three-dimensional conformal radiation therapy ((3D-CRT (60 Gy/30 f))	Mean dose of iSVZ greater than 57.9 Gy was an independent factor of OS	[66]
Elicin et al.	60 patients	+	+	3D-CRT (60 Gy/30 f)	Higher cSVZ dose (> 59.2 Gy) had a negative effect on OS and PFS	[67]
Bender et al.	200 patients	+	+	IMRT	Ipsilateral or contralateral SVZ dose had no significant effect on OS and PFS	[68]
Weinberg et al.	50 patients	+	+	External beam radiation therapy (60 Gy/30 f)	Distant SVZ site receiving ≤ 45 Gy had the shortest survival	[22]
Mathew et al.	47 patients	+	+	+	iSVZ dose ≥ 56 Gy trended toward improved OS and PFS, cSVZ dose ≥ 50 Gy appeared to have better OS and PFS	[73]

*SVZ* subventricular zone, *PFS* progression-free survival, *OS* overall survival, *iSVZ* ipsilateral SVZ, *GBM* glioblastoma, *GTR* gross total resection, *IMRT* intensity-modulated radiation therapy, *PTV* planning target volume, *3D-CRT* three-dimensional conformal radiation therapy, *cSVZ* contralateral SVZ; + receive treatment

The data from above-mentioned studies support the hypothesis that higher doses are needed to eliminate potential GBM cells in SVZ. However, the dose of ipsilateral and contralateral SVZ (cSVZ) has different effects on PFS and OS. Chen et al. [52] found that when the bilateral SVZ dose was greater than 40 Gy while the cSVZ dose was greater than 30 Gy, PFS could be improved. A study has shown that an ipsilateral mean dose of SVZ greater than 57.9 Gy was an independent factor of OS, while the same high dose of cSVZ had an opposite effect on survival [66]. Elicin et al. [67] seemed to confirm it. Their study has been found that higher cSVZ dose (> 59.2 Gy) had a negative effect on OS and PFS. In these retrospective studies, patients with higher cSVZ dose had poorer PFS, which may be associated with the large size of the tumor, crossing the midline, and local resection. And higher doses can cause neurocognitive toxicity and radioactive necrosis. The latest study by Katja Bender et al. found that iSVZ or cSVZ dose had no significant effect on OS and PFS. They suggested that the higher mean SVZ dose was a result of tumor central location and larger tumor size [68].

## Perspective overview in future clinical practice of SVZ as a therapeutic target for GBM

### Balance between the risks and benefits of radiotherapy in SVZ

Although scientists have made considerable efforts in the field of SVZ-based radiotherapy for GBM, the potential benefits resulting from the exposure of SVZ to radiation continue to be the focus of intense debate and significant controversy. As some studies have confirmed above, NSCs in SVZ are closely related to the occurrence, recurrence, and prognosis of GBM. Based on these studies, it can be supposed that the exposure of SVZ to radiation may eliminate GSCs, thus reduce the possibility of GBM recurrence and affect the prognosis of GBM patients.

However, we cannot neglect those retrospective studies that have shown unfavorable evidence not supporting the application of SVZ as a therapeutic target for GBM. Indeed, although the volume of SVZ is small, radiation to additional SVZ field except conventional clinical target volume of GBM may induce greater adverse effects. Especially for GBM far from the SVZ, it may result in more normal brain tissue being irradiated if the current focal radiation fields were to expand to include the SVZ. As significant neurocognitive deficits have been known to be associated with whole-brain irradiation [69], impairment of some neurocognitive functions would be expected. Furthermore, stem cells are notoriously difficult to treat with radiation, and higher doses may be required to achieve desired therapeutic effect. Since SVZ-NSCs are physiologically involved in the replenishment and repair of damaged nerve tissue, radiation damage to NSCs in SVZ may affect the repair ability of nerve function. Therefore, SVZ radiotherapy is a double-edged sword, and clinicians need to balance the interests.

There are some uncertain factors which affected the balance evaluation between beneficial effects and potential risk of the SVZ radiation for GBM patients. Therefore, the benefit of SVZ exposure to radiation in GBM treatment is still a challenging issue. Moreover, future research is critically needed to identify which GBM patients can be the beneficiaries of SVZ irradiation.

### The optimal modality of radiotherapy in SVZ

Since the location of primary tumor varies considerably among GBM patients, the radiation modality including radiation dose and radiation field required for SVZ radiotherapy may be different.

The current studies on SVZ radiotherapy are retrospective analysis, with certain data bias. Moreover, in these studies, the radiotherapy dose of SVZ is related to the location and volume of the tumor. The higher mean SVZ dose is a result of tumor central location and larger tumor size. The central location and large size reduce the chance of GTR. This relationship between SVZ dose, location, and volume may obfuscate the potential positive effect of SVZ irradiation in retrospective data. Secondly, there has not been a clear conclusion on the radiation field, radiotherapy mode, radiotherapy dose, segmentation method of SVZ and whether to irradiate cSVZ. Currently, some clinical data indicate that ipsilateral high-dose radiotherapy for SVZ can achieve better PFS. Iuchi et al. [64] mentioned in their study that low-partitioned high-dose radiotherapy can improve the survival of GBM patients, but they seem to take little consideration of adverse reactions. Theoretically, this type of irradiation can also increase the poor prognosis of patients. In addition, the radiation field should be different for GBM patients contacting to SVZ and GBM patients far from SVZ. GBM patients contacting to SVZ may improve PFS and even OS when exposed to high-dose radiation in SVZ, while irradiation of SVZ may be counterproductive in GBM patients far from SVZ. Whether irradiation is necessary for both iSVZ and cSVZ should be carefully determined depending on the tumor location to balance the advantages and disadvantages of radiating SVZ. Therefore, prospective, randomized studies of tumor bed combined with SVZ irradiation are needed.

### Challenge of other agents penetrating into SVZ

Due to the existence of blood–brain barrier and blood-cerebrospinal fluid barrier, the effect of conventional chemotherapy, immunotherapy, and targeted therapy on GBM is not good, and a single targeted drug is very easy to drug resistance. Based on several animal studies, injection of normal NSCs into the body or delivery of drugs with normal NSCs can influence the growth, invasion, and migration of GSCs, thereby improving the prognosis of GBM. Although there is not enough clinical evidence to support the safety and efficacy of this approach, some interesting clinical trials are underway. A phase 1 clinical study confirmed that NSC-delivered oncolytic adenovirus was safe and effective for the treatment of newly diagnosed gliomas [70]. This experiment took advantage of the direct oncolytic effect of oncolytic adenovirus and the ability to induce immune responses, combined with the ability of NSCs to cross the blood–brain barrier and migrate to tumor cells, and repeated injection of NSC-delivered oncolytic adenovirus into the resection margin wall in multiple directions during surgery, combined with postoperative radiotherapy and temozolomide chemotherapy, achieved good survival results. This study also has some limitations. This is an uncontrolled phase 1 clinical study. The good survival outcome of patients may be the result of earlier initiation of chemoradiotherapy. Larger cohort and better-grouped phase 2/3 clinical trials are needed to confirm the survival outcome. In addition, gene-modified NSCs in combination with some chemotherapy drugs are also being tried (*NCT02015819, NCT01172964, NCT02192359)*. Encouraging results from these studies are expected. Moreover, the site of transplantation, the method of administration and the concentration of the drug will all be considered in these studies, because all of which affect the specific immune response of the body [71].

## Conclusion

Over the years, both bench and bedside evidences strongly support the view that the presence of NSCs and GSCs in the SVZ may be the crucial factor of recurrence of GBM after conventional therapy. It emphasizes the necessity to explore new therapy strategies with the aim to target SVZ to eradicate NSCs or GSCs. Although previous studies indicated that preventive irradiation of SVZ may provide a new possibility for improving the prognosis of patients with GBM, there are still challenges in translating the preclinical data to clinical application. Considering the risks and benefits of radiotherapy in SVZ, a well-designed prospective clinical trial revolving the radiation dose and radiation field is needed to establish the safety and feasibility of this strategy. Moreover, it needs to be carefully considered by clinicians that which group of people will benefit most from SVZ prophylactic radiation by searching for sensitive molecular markers. In addition, the use of NSCs in SVZ as GBM therapeutic targets for targeted agents therapy also needs to be further confirmed by experimental and clinical studies.

Taken together, although there remain unresolved issues, current advances provide us with a lot of evidence that targeting the NSCs and GSCs in SVZ may have the potential to solve the dilemma of GBM recurrence and treatment resistance. We hope that these benefits of targeting the NSCs and GSCs in SVZ will be achieved soon and become commonplace in the treatment of GBM. This could substantially change the conventional treatment modality of GBM.

## Data Availability

Not applicable.

## Abbreviations

GBM: Glioblastoma
GSCs: Glioma stem cells
SVZ: Subventricular zone
NSCs: Neural stem cells
NPCs: Neural precursor cells
OS: Overall survival
CTX: Cortex
MRI: Magnetic resonance imaging
CSCs: Cancer stem cells
PTN: Pleiotrophin
EGFR: Epidermal growth factor receptor
VEGF: Vascular endothelial growth factor
PFS: Progression-free survival
iSVZ: Ipsilateral SVZ
PTV: Planning target volume
CTV: Clinical target volume
cSVZ: Contralateral SVZ
IMRT: Intensity-modulated radiation therapy
GTR: Gross total resection
STR: Subtotal resection
3D-CRT: Three-dimensional conformal radiation therapy
